# Global insights into MRSA bacteremia: a bibliometric analysis and future outlook

**DOI:** 10.3389/fmicb.2024.1516584

**Published:** 2025-01-22

**Authors:** Jia-Yi Lin, Jia-Kai Lai, Jian-Yi Chen, Jia-Yu Cai, Zhan-Dong Yang, Liu-Qingqing Yang, Ze-Tao Zheng, Xu-Guang Guo

**Affiliations:** ^1^Department of Clinical Laboratory Medicine, Guangdong Provincial Key Laboratory of Major Obstetric Diseases, Guangdong Provincial Clinical Research Center for Obstetrics and Gynecology, The Third Affiliated Hospital, Guangzhou Medical University, Guangzhou, China; ^2^The Second School of Clinical Medicine, Guangzhou Medical University, Guangzhou, China; ^3^Department of Biomedical Engineering, The School of Biomedical Engineering, Guangzhou Medical University, Guangzhou, China; ^4^Department of Clinical Medicine, The Third School of Clinical Medicine, Guangzhou Medical University, Guangzhou, China; ^5^School of Medicine, South China University of Technology, Guangzhou, Guangdong, China; ^6^Guangdong Cardiovascular Institute, Guangdong Provincial People’s Hospital (Guangdong Academy of Medical Sciences), Southern Medical University, Guangzhou, Guangdong, China; ^7^Guangzhou Key Laboratory for Clinical Rapid Diagnosis and Early Warning of Infectious Diseases, King Med School of Laboratory Medicine, Guangzhou Medical University, Guangzhou, China

**Keywords:** MRSA, bloodstream infection, antibiotic resistance, artificial intelligence in diagnosis, personalized medicine

## Abstract

**Background:**

Methicillin-resistant *Staphylococcus aureus* (MRSA) bloodstream infections (BSIs) pose a significant challenge to global public health, characterized by high morbidity and mortality rates, particularly in immunocompromised patients. Despite extensive research, the rapid development of MRSA antibiotic resistance has outpaced current treatment methods, increasing the difficulty of treatment. Therefore, reviewing research on MRSA BSIs is crucial.

**Methods:**

This study conducted a bibliometric analysis, retrieving and analyzing 1,621 publications related to MRSA BSIs from 2006 to 2024. The literature was sourced from the Web of Science Core Collection (WoSCC), and data visualization and trend analysis were performed using VOSviewer, CiteSpace, and Bibliometrix software packages.

**Results:**

The bibliometric analysis showed that research on MRSA BSIs was primarily concentrated in the United States, China, and Japan. The United States leads in research output and influence, with significant contributions from institutions such as the University of California system and the University of Texas system. The journal with the most publications is Antimicrobial Agents and Chemotherapy, while the most cited global publication is Vincent JL’s article “Sepsis in European Intensive Care Units: Results of the SOAP Study” published in Critical Care Medicine in 2006. Cosgrove SE’s article “Comparison of Mortality Associated with Methicillin-Resistant and Methicillin-Susceptible *Staphylococcus aureus* Bacteremia: A Meta-analysis” had the most co-citations. Key trends in the research include MRSA’s antibiotic resistance mechanisms, the application of new diagnostic technologies, and the impact of COVID-19 on MRSA studies. Additionally, artificial intelligence (AI) and machine learning are increasingly applied in MRSA diagnosis and treatment, and phage therapy and vaccine development have become future research hotspots.

**Conclusion:**

Methicillin-resistant *Staphylococcus aureus* BSIs remain a major global public health challenge, especially with the increasing severity of antibiotic resistance. Although progress has been made in new treatments and diagnostic technologies, further validation is required. Future research will rely on integrating genomics, AI, and machine learning to drive personalized treatment. Strengthening global cooperation, particularly in resource-limited countries, will be key to effectively addressing MRSA BSIs.

## 1 Introduction

Methicillin-resistant *Staphylococcus aureus* (MRSA) bloodstream infections (BSIs) are a major public health challenge worldwide (Brown et al., 2021; Mahmoud et al., 2020). As a pathogen resistant to ß-lactam antibiotics, MRSA has become a leading cause of hospital- and community-acquired infections (Kavanagh, 2019), and MRSA BSIs are often associated with high morbidity and mortality, particularly in immunocompromised patients (Hassoun et al., 2017). Despite ongoing research, the development of resistance to MRSA continues to outpace advances in available therapies, making it increasingly difficult to treat (Evans et al., 2021). The impact of MRSA infections on healthcare systems worldwide has prompted scientists to work toward understanding the mechanisms of resistance, the routes of transmission and the search for alternative therapeutic approaches (Brown et al., 2021).

Previous studies have highlighted the increasing incidence of MRSA BSIs and their impact on the healthcare environment (Popovich et al., 2021). In particular, the ability of MRSA to spread in high-risk areas such as intensive care units (ICUs) has been extensively studied (Kavanagh, 2019). Although antibiotics such as vancomycin and daptomycin have shown some efficacy in treatment, the resulting problem of drug resistance is increasing, leading to a gradual decline in therapeutic efficacy (Evans et al., 2021). In addition, delayed diagnosis is a key factor contributing to increased mortality, and early detection and timely treatment are critical to the survival of patients with MRSA BSIs (Brown et al., 2021). In recent years, new technologies such as whole-genome sequencing (WGS) and machine learning (ML) have opened new avenues for early detection and improved treatment (Evans et al., 2021).

However, there are still many gaps in current research into MRSA BSIs. In particular, there is a lack of in-depth understanding of the evolution of resistance patterns in different regions and the effectiveness of available treatments. In addition, although international collaborations have produced some results in mapping the global distribution of MRSA (Stefani et al., 2012; Tokajian, 2014), the role of multi-institutional collaborations and new diagnostic technologies (Gammel et al., 2021; Tang et al., 2022) in the management of MRSA has not been fully explored. A thorough understanding of these issues is essential for the development of effective global response strategies.

This study aims to comprehensively explore research trends in the field of MRSA BSIs through a bibliometric analysis of studies conducted between 2006 and 2024. Bibliometrics (Li et al., 2024) is a strategy used to investigate library and data science by conducting a quantitative analysis of bibliographic data. It helps researchers quickly understand their areas of interest and emerging trends, thereby laying a foundation for future research directions. Given the current lack of research and summaries on the future trends of MRSA BSIs, we conducted a bibliometric study based on published data in this field. We hope this will contribute to the development of MRSA BSI research and provide future researchers with guidance for broader and more effective applications in the future.

## 2 Materials and methods

### 2.1 Data sources and search strategies

**FIGURE 1:**
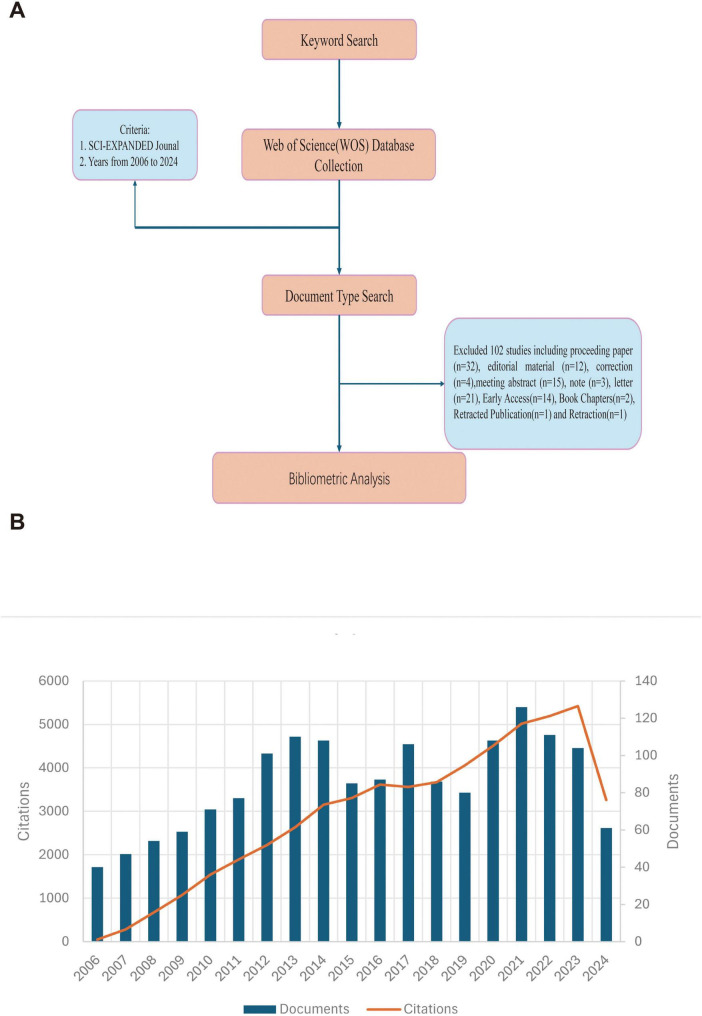
**(A)** A process flowchart. **(B)** The number of annual publications and citations of blood infection of MRSA research from 2006 to 2024 has been steadily increasing.

The Web of Science Core Collection (WoSCC) was searched for articles on MRSA and BSIs from 2006 to 2024. The literature search was completed in 1 day (24 August 2024) to eliminate citation oscillations caused by rapid publication updates. The search formula was set as ((TS = (MRSA)) OR TS = (Methicillin Resistant *Staphylococcus aureus*) AND (TS = (bloodstream infection) OR TS = (Bloodstream Infections) OR TS = (Infection, Bloodstream) OR TS = (Infection, Bloodstream) OR TS = (Septicemia) OR TS = (Septicemias) OR TS = (Blood Poisoning) OR TS = (Blood Poisonings) OR TS = (Poisonings, Blood) OR TS = (Severe Sepsis) OR TS = (Poisoning, Blood) OR TS = (Sepsis, Severe) OR TS = (Pyemia) OR TS = (Pyemias) OR TS = (Pyaemia) OR TS = (Pyaemias) OR TS = (Pyohemia) OR TS = (Pyohemias)). The only publication types used were Article and Review; in addition, the results of the study were documented in “plain text” format in the content of the “Full Record and Cited References.” The file contains: (i) author(s), (ii) abstract, (iii) address, (iv) ISSN/ISBN, (v) IDS number, (vi) funding information, (vii) PubMed ID, (viii) title, (ix) cited reference(s), (x) number of citations, (xi) count of cited references, (xii) language, (xiii) source, ((xiv) document type, (xv) keyword, (xvi) source abbreviation, (xvii) author identifier, (xviii) article information, (xix) publisher information, (xx) field of study, (xxi) usage count, and (xxii) high citation count. Finally, 1,621 original articles or reviews were included in the bibliometric analysis. Figure 1A depicts the detailed process.

### 2.2 Data analysis

All valid data were imported into Microsoft Office Excel 2019, the R-based package Bibliometrix 4.3.3, VOSviewer (version 1.6.20) and CiteSpace (version 6.3.R1) for visualization and analysis.

VOSviewer is another bibliometric analysis software developed by Nees Jan van Eck and Ludo Waltman for web-based data construction and viewing of bibliometric maps, from which key information can be extracted from a wide range of publications (10) (van Eck and Waltman, 2010). It allows the creation of visual representations of bibliometric networks, including collaboration between countries and institutions. The different clusters are indicated by the color of the nodes, the number of publications by the size of the nodes, and the strength of the relationships by the thickness of the lines.

CiteSpace, developed by Professor Chaomei Chen, is a general-purpose JAVA-based program for bibliometric and visual analysis (11) (Chen, 2004). CiteSpace is used to perform cluster analysis, create bursts of citations and keywords, and timeline views. By categorizing the keywords and references, cluster analysis of keywords can reveal the key areas of MRSA blood infection. Silhouette >0.5 and modularity *Q* > 0.3 indicate that the clustering results are adequate and convincing. The clustering of keywords and references can be used to discover new trends in MRSA blood infection research.

Bibliometrix packages is a bibliometric analysis tool based on the R programming language for mathematical and statistical calculations of publication frequency, percentage and number of citations for each journal, country, etc.

## 3 Result

### 3.1 Trends in publications and citations

According to our research strategy, a total of 1,621 publications related to blood infection of MRSA from 2006 to 2024 were obtained from the WoSCC database. Figure 1B illustrates the annual number of publications and citations for blood infection of MRSA research from 2006 to 2024. Thus, in 2006, the field of research was already well established. The steady increase in publications from 2006 to 2013 reflects the tremendous attention and strong interest in this area of research, but after 2014, there is an overall steady trend in the number of publications, suggesting that research on blood infection of MRSA continues to be hot but not explosive. The trend of increasing citations also suggests that research in this area is not only numerous but also far-reaching, continuing to attract scholars to conduct research related to blood infection of MRSA, and that more prospective research is needed in this area in the future, highlighting its global relevance.

### 3.2 National and institutional analyses

**FIGURE 2:**
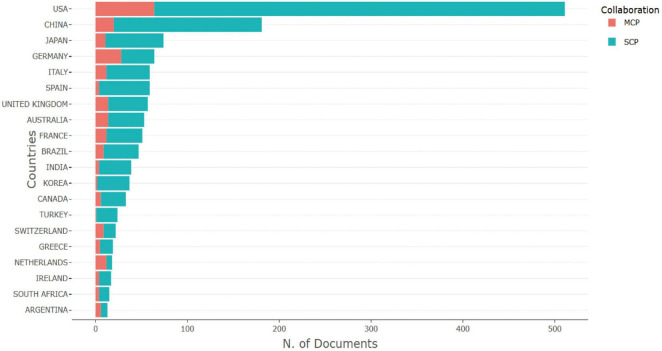
Top 20 most corresponding author’s country in the blood infection of MRSA domain.

A total of 111 countries and 2,726 institutions were involved in research on MRSA BSIs, and the 10 most prolific countries in terms of NP- and TC-based publications on MRSA BSIs were ranked (Table 1), with the United States leading the way in research on MRSA BSIs, with a total of 511 publications, followed by China (181) and Japan (74). The United States also had the highest total number of citations, highlighting its influence in this area of research. Multinational publications (MCP) describes the proportion of publications in a specific field that involve contributions from multiple countries, and it is used to evaluate the level of international collaboration of a country in a particular research domain. It is worth noting that Germany and the Netherlands have a high percentage of MCP (Figure 2), suggesting a significant contribution to international collaboration. Although the UK has fewer publications, it has very close research exchanges with other countries (Table 1).

**TABLE 1:** The top 10 productive countries with publications concerning blood infection of MRSA.

Rank	Countries	NP	SCP	MCP	MCP%	Total link strength	Countries	TC
1	USA	511	447	64	12.5	203	USA	26,431
2	China	181	161	20	11	64	China	3,137
3	Japan	74	63	11	14.9	22	Germany	2,430
4	Germany	64	36	28	43.8	128	Belgium	2,330
5	Italy	59	47	12	20.3	94	Canada	1,911
6	Spain	59	55	4	6.8	68	United Kingdom	1,770
7	United Kingdom	57	43	14	24.6	136	Australia	1,682
8	Australia	53	39	14	26.4	46	Spain	1,626
9	France	51	39	12	23.5	101	Italy	1,339
10	Brazil	47	38	9	19.1	50	France	1,217
11	USA	511	447	64	12.5	203	USA	26,431

NP, number of publications; SCP, single country publication; MCP, multiple country publication; TC, total citation.

**FIGURE 3:**
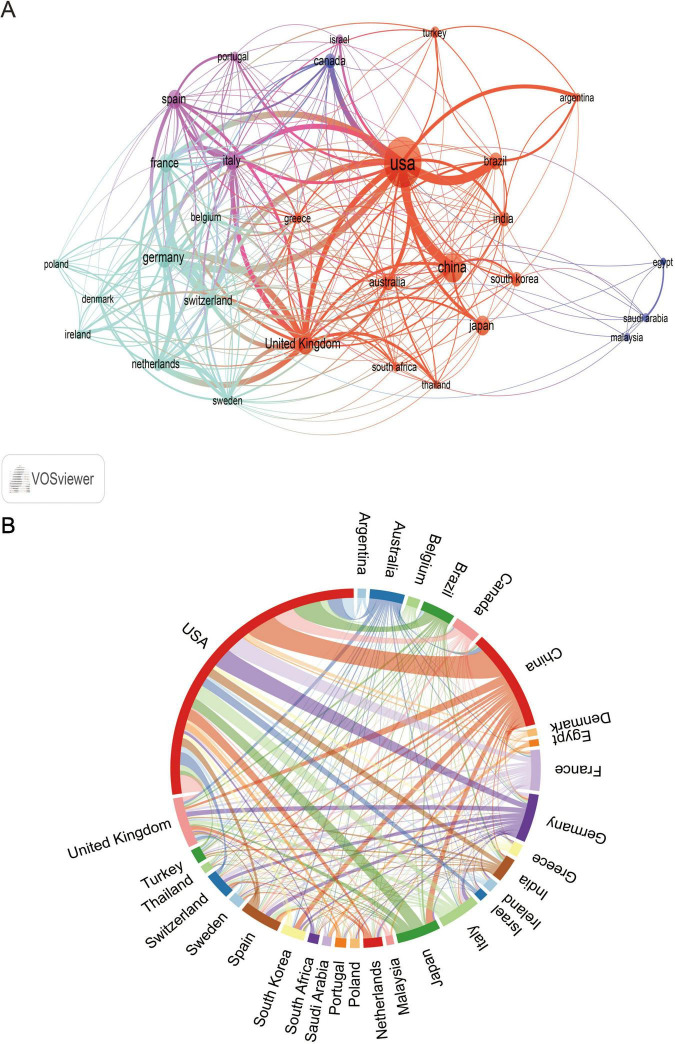
The analysis of countries related to blood infection of MRSA. **(A)** Co-authors–countries collaboration visualization in blood infection of MRSA. **(B)** The network map of cooperation between countries.

A minimum number of articles of 13 was set to filter out the 30 countries that met the set parameters, and the close cooperation between the countries is shown in Figures 3A, B, which shows the extensive network of cooperation between the countries, with the United States, China, and the European countries forming the key hubs in the global research network. Figure 3B further confirms the prominence of these countries in terms of the number of collaborations and publications between them in this research area.

Among the top 10 institutions in terms of number of publications, the University of California system is the most productive institution with 116 publications and 3,217 total citations, followed by the University of Texas system (NP: 71) and Harvard University (NP: 66) (Table 2). Not only do these institutions contribute significantly in terms of publication volume, they are equally significant in terms of impact. Among them, although ASSISTANCE PUBLIQUE HOPITAUX PARIS (APHP) is only ranked fourth in terms of the number of publications (NC: 61), it is ranked second in terms of the total number of citations (TC: 2,782), which indicates that the quality and impact of its publications are very high.

**TABLE 2:** Top 10 central institutions studying the blood infection of MRSA.

Rank	Institution	NP	TC	Countries
1	University of California System	116	3,217	USA
2	University of Texas System	71	1,867	USA
3	Harvard University	66	1,614	USA
4	Assistance Publique Hopitaux Paris (APHP)	61	2,782	France
5	National Taiwan University	59	641	China
6	Wayne State University	58	1,502	USA
7	US Department of Veterans Affairs	57	1,875	USA
8	Veterans Health Administration (VHA)	56	1,778	USA
9	Duke University	55	1,180	USA
10	University of California Los Angeles	55	1,049	USA

NP, number of publications; TC, total citation.

**FIGURE 4:**
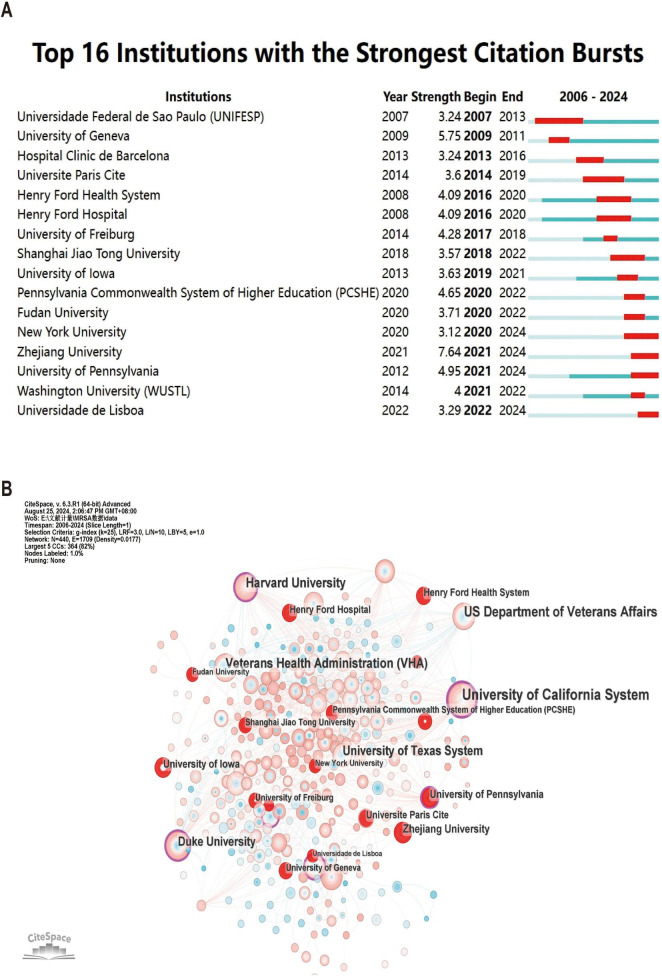
The analysis of research institutions related to blood infection of MRSA. **(A)** Co-authors–institute collaboration visualization in blood infection of MRSA. **(B)** The co-occurrence map of research institutions. The node size means the co-occurrence frequencies, while the linkages mean the co-occurrence relationships. Nodes with purple round mean high betweenness centrality (≥0.1).

Figure 4A illustrates the top 16 institutions with citation outbreaks, with Zhejiang University and New York University experiencing citation outbreaks in the recent past, suggesting that they have a lot of potential in the field of MRSA BSIs today. Co-occurrence graph (Figure 4B) Node size indicates co-occurrence frequency, while links indicate co-occurrence relationships. Nodes with purple rounded corners indicate high mediator centrality (≥0.1). The critical role of institutions with high mediator centrality (e.g., the University of California system) in connecting diverse research communities.

### 3.3 Analysis of journals

**FIGURE 5:**
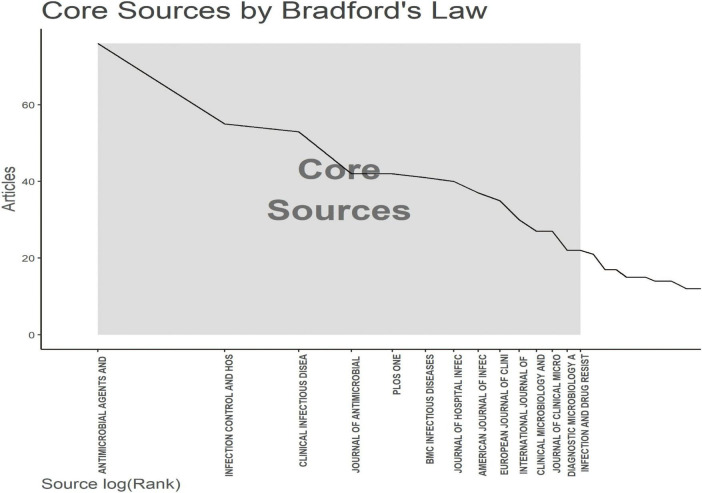
The analysis of academic journals related to blood infection of MRSA. Bradford’s law according to the academic journals.

To show active and influential journals on MRSA BSIs, a visual analysis of published journals was conducted. We found 1,621 publications related to MRSA BSIs published in 494 academic journals. Table 3 and Figure 5 show that the journal with the most publications was ANTIMICROBIAL AGENTS AND CHEMOTHERAPY (NC: 76), followed by INFECTION CONTROL AND HOSPITAL EPIDEMIOLOGY (NC: 55), and CLINICAL INFECTIOUS DISEASES (NC: 53). In addition, CLINICAL MICROBIOLOGY AND INFECTION has the highest impact factor (10.9) among the top 10 journals, but his impact in the field of MRSA BSIs is not exceptional.

**TABLE 3:** The top 10 influential academic journals with publications concerning blood infection of MRSA.

Rank	Source	h_index	NP	TC	IF
1	Antimicrobial Agents and Chemotherapy	36	76	4,708	4.1
2	Clinical Infectious Diseases	34	53	4,420	8.2
3	Journal of Antimicrobial Chemotherapy	26	42	1,555	3.9
4	Infection Control and Hospital Epidemiology	25	55	4,631	3.0
5	PLoS One	25	42	1,518	2.9
6	Clinical Microbiology and Infection	21	27	1,808	10.9
7	Journal of Clinical Microbiology	20	27	1,349	6.1
8	American Journal of Infection Control	18	37	1,549	3.8
9	BMC Infectious Diseases	17	41	740	3.4
10	International Journal of Antimicrobial Agents	17	30	782	4.9

NP, number of publications; TC, total citation; h_index, Hirsch index; IF, impact factor.

Figure 5 applies Bradford’s law to show the core journals in MRSA BSIs research.

### 3.4 Author contributions and co-occurrence

**FIGURE 6:**
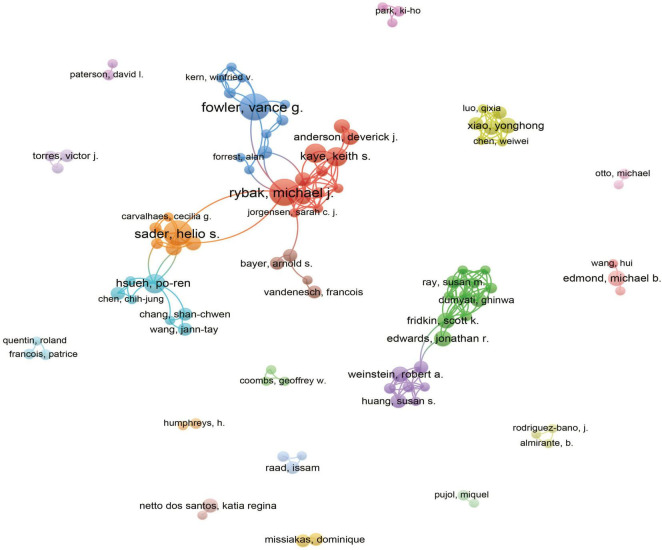
The analysis of authors related to blood infection of MRSA. The co-occurrence authors’ map of blood infection of MRSA research. The varied colored nodes 766 reflect the authors in various clusters. The node size means the co-occurrence frequencies, while the 767 linkages mean the co-occurrence relationships between authors.

A total of 9,425 authors were involved in the study of MRSA BSIs. Table 4 shows the top 10 most relevant authors in MRSA BSI research. We can see that Fowler VG leads with 16 articles and 867 citations, followed by Rybak MJ with 17 articles and 507 citations. It is worth noting that Lodise TP has a small number of articles but the largest number of citations, which shows that his articles are very influential in the field. The interconnectedness of these authors is further illustrated by the co-occurrence diagram in Figure 6, showing the collaborative network between authors that provides expert information for finding research partners, with the 21 colors representing the 21 clusters in Figure 6. Fowler, Vance G. and Rybak, Michael J. are at the heart of the collaborative network. Authors actively collaborate on MRSA BSIs, especially between authors in the same cluster, e.g. Kaye, Keith S. and Rybak, Michael J. Close co-operation between authors from different clusters can also be observed, e.g., Fowler, Vance G. and Kern, Winfried V.

**TABLE 4:** Top 10 most relevant authors and their production.

Rank	Author	h_index	NP	TC
1	Fowler VG	12	16	867
2	Rybak MJ	12	17	507
3	Sader HS	12	17	779
4	Humphreys H	10	11	447
5	Kaye KS	10	12	427
6	Lodise TP	10	14	1,298
7	Anderson DJ	9	10	439
8	Dumyati G	9	9	950
9	Edmond MB	9	10	494
10	Jones RN	9	12	637

NP, number of publications; TC, total citation; h_index, Hirsch index.

### 3.5 Citation and reference analyses

Table 5 shows the top 10 most cited articles on MRSA BSIs, with the top 4 articles having more than 1,000 citations. The most cited article globally was Vincent JL’s article “Sepsis in European intensive care units: results of the SOAP study” published in Critical Care Medicine in 2006 with 1,954 citations (Vincent et al., 2006). In this article, MRSA was firstly introduced as one of the important pathogens leading to sepsis, which laid the foundation for subsequent studies on MRSA BSIs.

**TABLE 5:** Top 10 most globally cited documents concerning blood infection of MRSA.

Rank	Title	First author	Journal	Year	TC
1	Sepsis in European intensive care units: results of the SOAP study*	Vincent JL	Critical Care Medicine	2006	1,954
2	Antimicrobial-resistant pathogens associated with healthcare-associated infections: annual summary of data reported to the National Healthcare Safety Network at the Centers for Disease Control and Prevention, 2006–2007	Hidron AI	Infection Control & Hospital Epidemiology	2008	1,556
3	Community-associated methicillin-resistant *Staphylococcus aureus*: epidemiology and clinical consequences of an emerging epidemic	David MZ	Clinical Microbiology Reviews	2010	1,420
4	Community-associated methicillin-resistant *Staphylococcus aureus*: epidemiology and clinical consequences of an emerging epidemic	Sievert DM	Infection Control & Hospital Epidemiology	2013	1,181
5	Emergence of community-associated methicillin-resistant *Staphylococcus aureus* USA300 genotype as a major cause of health care—associated blood stream infections	Seybold U	Clinical Infectious Diseases	2006	544
6	Targeted versus universal decolonization to prevent ICU infection	Huang SS	The New England Journal of Medicine	2013	542
7	Pathogenicity and virulence of *Staphylococcus aureus*	Cheung GYC	Virulence	2021	519
8	Relationship between vancomycin MIC and failure among patients with methicillin-resistant *Staphylococcus aureus* bacteremia treated with vancomycin	Lodise TP	Antimicrobial Agents and Chemotherapy	2008	454
9	*Vital signs:* epidemiology and recent trends in methicillin-resistant and in methicillin-susceptible *Staphylococcus aureus* bloodstream infections – United States	Kourtis AP	Morbidity and Mortality Weekly Report	2019	439
10	Molecular mechanisms of NET formation and degradation revealed by intravital imaging in the liver vasculature	Kolaczkowska E	Nature Communications	2015	421

TC, total citation.

The co-cited references (Table 6) highlight the foundational studies by authors such as Cosgrove et al. (2003) and Liu et al. (2011), which are often cited together, demonstrating their important role in the literature. Table 6 shows the top 10 co-cited papers related to MRSA BSIs and shows the number of times each paper was cited. Of these, four articles were cited more than 100 times. The most cited article was COSGROVE SE’s “Comparison of Mortality Associated with Methicillin-Resistant and Methicillin-Susceptible *Staphylococcus aureus* Bacteremia: a Meta-analysis,” published in the journal Clinical Infectious Diseases in 2003 (Cosgrove et al., 2003). This article introduced MRSA BSIs and laid the foundation for subsequent research on bacteremia. Liu et al. (2011) published in the journal Clinical Infectious Diseases “Clinical Practice Guidelines by the Infectious Diseases Society of America for the Treatment of Methicillin-Resistant *Staphylococcus aureus* Infections in Adults and Children,” was cited 125 times.

**TABLE 6:** The top 10 co-cited references concerning blood infection of MRSA.

Rank	Title	First author	Journals	Year	TC
1	Comparison of mortality associated with methicillin-resistant and methicillin-susceptible *Staphylococcus aureus* bacteremia: a meta-analysis	Cosgrove SE	Clinical Infectious Diseases	2003	140
2	Clinical practice guidelines by the Infectious Diseases Society of America for the treatment of methicillin-resistant *Staphylococcus aureus* infections in adults and children	Liu C	Clinical Infectious Diseases	2011	125
3	Invasive methicillin-resistant *Staphylococcus aureus* infections in the United States	Klevens RM	Journal of the American Medical Association	2007	109
4	Antimicrobial-resistant pathogens associated with healthcare-associated infections: annual summary of data reported to the National Healthcare Safety Network at the Centers for Disease Control and Prevention, 2006–2007	Horan TC	American Journal of Infection Control	2008	107
5	Multilocus sequence typing for characterization of methicillin-resistant and methicillin-susceptible clones of *Staphylococcus aureus*	Enright MC	Journal of Clinical Microbiology	2000	99
6	*Staphylococcus aureus* infections	Lowy FD	The New England Journal of Medicine	1998	93
7	CDC definitions for nosocomial infections, 1988	Garner JS	American Journal of Infection Control	1988	85
8	Evolutionary dynamics of *Enterococcus faecium* reveals complex genomic relationships between isolates with independent emergence of vancomycin resistance	Van Hal SJ	Clinical Microbiology Reviews	2012	84
9	Influence of vancomycin minimum inhibitory concentration on the treatment of methicillin-resistant *Staphylococcus aureus* bacteremia	Soriano A	Clinical Infectious Diseases	2008	81
10	The Charlson comorbidity index is adapted to predict costs of chronic disease in primary care patients	Charlson ME	Journal of Chronic Diseases	1987	80

TC, total citation.

**FIGURE 7:**
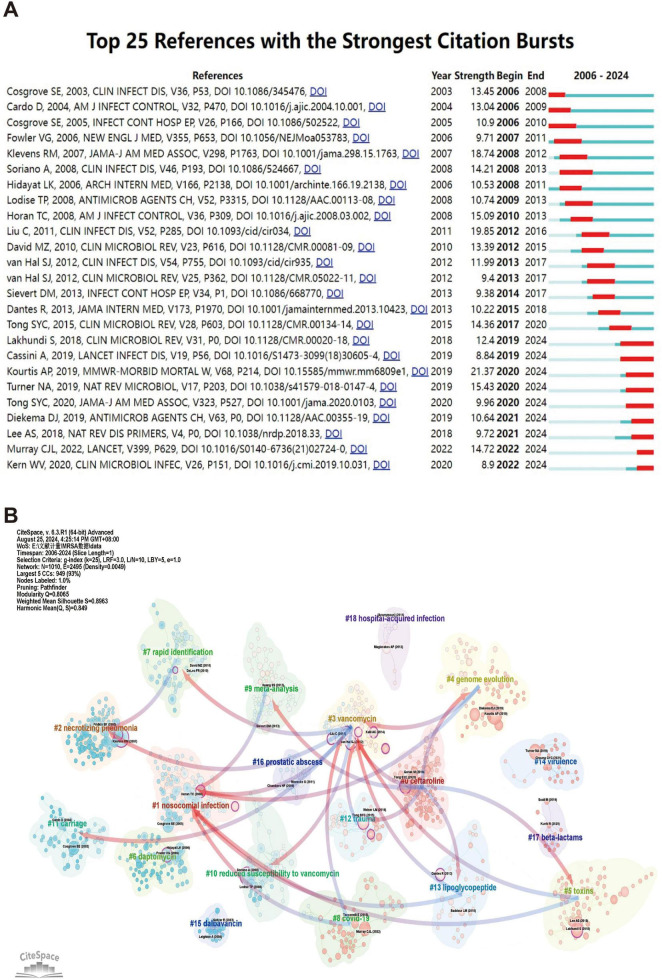
The analysis of references related to blood infection of MRSA. **(A)** The top 25 references with strong citation bursts. A red bar means high citations in the corresponding year. **(B)** Clustering of references based on the similarity between references, including #0 ceftaroline, #1 nosocomial infection, #2 necrotizing pneumonia, #3 vancomycin, #4 genome evolution, #5 toxins, #6 daptomycin, #7 rapid identification, #8 COVID-19, and so on. While linkages refer to the connections that develop between different clusters, and the blue groups on the line evolve from the red ones.

Figure 7A illustrates the 25 core literatures with a significant increase in citation frequency, reflecting the significant impact and cutting edge of these literatures on the relevant research field in a given time period. Earlier literature, such as Cosgrove et al. (2003) and Cardo (2004), experienced a strong citation explosion between 2006 and 2010, suggesting that they featured prominently in the topics of nosocomial infection control, antimicrobial drug use, and so on, during that period. These literatures laid the foundation for research on antimicrobial resistance and infection prevention, providing a theoretical framework and empirical support for subsequent studies.

As the study continued, subsequent literature, such as Liu et al. (2011) and David and Daum (2010), saw a significant increase in citation frequency between 2012 and 2016. This period coincided with a surge in global interest in drug-resistant microbial infections and antimicrobial stewardship, and Liu et al.’s (2011) study has had a profound impact on the clinical infection field, providing valuable insights into the rational use of antibiotics and mechanisms of resistance, while David and Daum’s (2010) study has further explored the link between community-acquired and hospital-acquired infections, advancing the field of microbial drug resistance. David and Daum’s (2010) study further explored the link between community-acquired infections and hospital-acquired infections and contributed to the in-depth research in the field of microbial resistance.

In recent years, some of the more recent literature has begun to spark a sustained citation explosion post-2020 as the problem of emerging infectious diseases and antibiotic resistance intensifies. For example, literature such as Kourtis et al. (2019) and Turner et al. (2019) experienced significant citation growth between 2020 and 2024, demonstrating its importance in the context of the COVID-19 pandemic. Kourtis et al.’s (2019) research on emerging infectious diseases provided key insights into outbreak response strategies, while Turner et al.’s (2019) work on provide insights into the mechanisms of transmission of drug-resistant bacteria. In addition, the study by Murray et al. (2022) reflects changes in the global health burden during an outbreak, and its findings are widely used in current public health decision-making, making it a cutting-edge literature in the field.

The use of co-citation cluster analysis provides an objective indication of the knowledge structure of the research area. To further describe the co-cited reference groups, we created a network diagram. The degree of association between articles is divided into 18 categories, which are the basis of the cluster classification. In the diagram, the co-citation cluster analysis clearly reveals the knowledge structure of the research area. In order to fully describe the co-cited literature groups, we constructed a complex network diagram (shown in Figure 7B). Research topics were classified into 18 categories based on co-citation relationships, forming a clear cluster structure. One nosocomial infection is the largest cluster in the diagram, indicating that nosocomial infection-related research occupies a central position in the field and has broad academic influence. This cluster is closely linked to three vancomycin by multiple connecting lines, suggesting that studies of vancomycin treatment and nosocomial infections co-occur frequently in the literature, demonstrating a strong correlation between the two.

Evolution between the study clusters over time is also evident. For example, eight the COVID-19 cohort evolved gradually from studies of the one nosocomial infection and three vancomycin cohorts, and nosocomial infection and antibiotic resistance studies have expanded further in the neo-crowning context, especially with the global epidemic of COVID-19. This demonstrates the intersection and correlation between emerging infectious diseases and traditional antibiotic resistance research. In addition, 10 reduced susceptibility to vancomycin groups also evolved from red groups (e.g., three vancomycin), and this evolution reflects the further refinement of antimicrobial drug resistance research, which has gradually shifted from the use of vancomycin to an in-depth exploration of its resistance mechanisms.

In addition, four genome evolution groups have gradually diverged from those associated with antimicrobial drug use (e.g., three vancomycin), evolving into the research field of pathogen genome evolution. This trend clearly indicates that with advances in genomics technology, researchers have begun to pay more attention to the mechanisms of genetic evolution of pathogens and have attempted to better understand changes in drug resistance and infectivity through genomic analyses.

In addition to these major evolutionary trends, a number of smaller groups reflect the continued development of specific research directions. For example, the 13 lipoglycopeptide group has evolved to target studies of resistance mechanisms to specific antimicrobial drugs through its close association with three vancomycin. This suggests that research is not only focusing on widely used antibiotics, but is also progressively expanding toward refined antibiotic class resistance.

### 3.6 Keywords and research area trends

Usually, keywords reflect the research topic and content of an article. By analyzing keywords, we can quickly understand the research trend in a certain field. We listed four common keywords for MRSA: infections, blood stream infections, methicillin resistance, and *Staphylococcus aureus*. These four main keywords were focused on the mechanism of MRSA resistance, the transmission route of the infection, and the prevention and treatment of related diseases.

**FIGURE 8:**
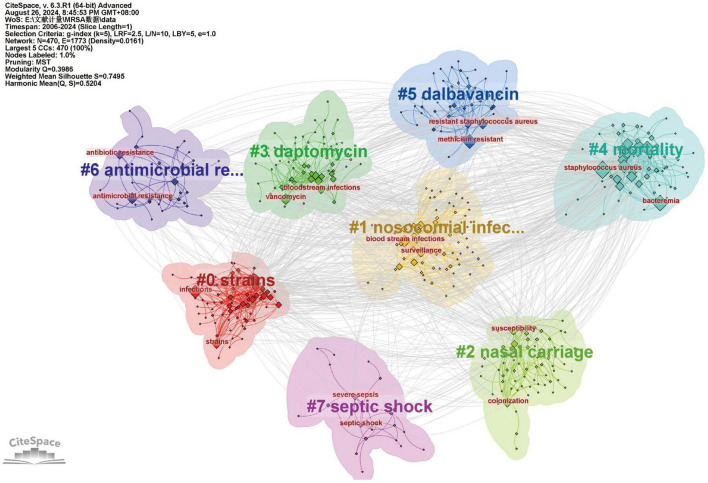
The analysis of research fields and keywords concerning blood infection of MRSA.

Subsequently, we performed keyword cluster analysis and obtained eight clusters (Figure 8). These eight clusters are as follows: #0 strains, #1 nosocomial infections, #2 nasal carriage, #3 daptomycin, #4 mortality, #5 dalbavancin, #6 antimicrobial resistance, #7 septic shock. #0 strains is the largest cluster, and the main keywords in the field are strains and infections.

**FIGURE 9:**
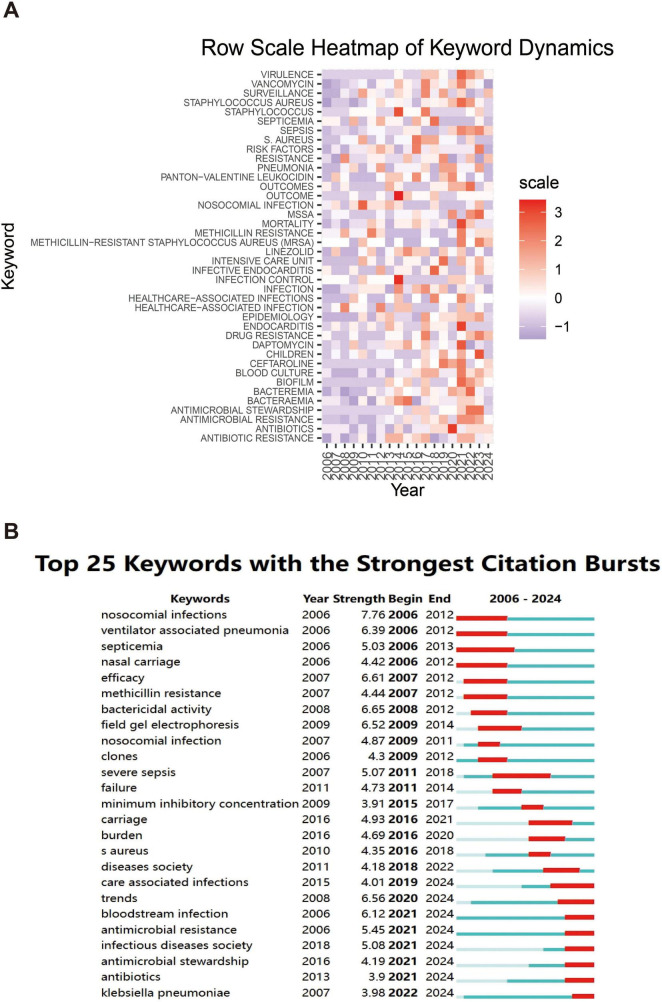
Heatmap analysis of keywords. **(A)** The annual heatmap related to blood infection of MRSA research. The annual popularity of the keyword is measured as the standardized frequency of a keyword’s occurrence in a given year. **(B)** The top 25 keywords with the strongest citation bursts.

The heatmap analysis in Figure 9A details the popularity of specific keywords across years, revealing the evolution of research topics over time. The color shades in the heatmap reflect the research intensity of the keyword, with red areas indicating high research intensity for the keyword in a given year and purple areas showing relatively low research activity. Keywords such as “antibiotic resistance,” “antimicrobial stewardship,” and “blood culture” are shown. “Blood culture” have come to the forefront of research in recent years. This suggests that as clinical needs change, research is expanding from the issue of antibiotic resistance to how to effectively respond to infections through antimicrobial stewardship and new diagnostic approaches.

Figure 9B further demonstrates the dynamics of research hotspots through the citation bursts of keywords. Keywords with strong citation bursts indicate an explosive growth of research in these areas over a specific time period. The figure shows that keywords such as, “antibiotics,” “antimicrobial resistance,” and “care associated infections” (care-associated infections) show a strong citation burst between 2021 and 2024, with research related to COVID-19 in particular further driving the intensity of research on these keywords. In addition, keywords related to “MRSA” BSIs, such as “methicillin resistance” and “clones” (clones), showed a high citation intensity between 2006 and 2012, indicating that the genome evolution study of MRSA attracted widespread attention at that time.

An overall analysis of the two graphs shows that the focus of infectious disease research is gradually expanding from the traditional pathogen resistance to more complex clinical management and diagnostic techniques, particularly driven by the recent outbreaks, where researchers have shown a great deal of interest in how to control nosocomial infections and the spread of drug-resistant strains.

## 4 Discussion

### 4.1 Overall distribution

From 2006 to 2024, research on MRSA BSIs has grown significantly. This growth reflects the global academic community’s high level of concern over MRSA bacteremia as a serious public health issue (Hassoun et al., 2017). MRSA bacteremia, defined as BSIs caused by MRSA, has extremely high morbidity and mortality rates (Jaganath et al., 2018), particularly in immunocompromised patients. Globally, the United States, China, and European countries are the primary contributors to this field, showcasing their research strengths in addressing the global threat posed by MRSA bacteremia. The United States undoubtedly leads in terms of research output in this area, with 511 related publications, demonstrating not only its vast research scale but also its global influence in MRSA BSI research. At the same time, China and Japan contributed 181 and 74 papers, respectively, indicating the growing importance of Asian countries in this field, particularly in their rapid rise in studying MRSA bacteremia.

Compared to past research on antimicrobial resistance, the global distribution trend in MRSA BSI research is similar to that of other BSIs caused by antibiotic-resistant pathogens. North America and Europe have historically been the major drivers of antibiotic resistance research, with their research institutions and academic journals playing crucial roles in global scientific progress. Key institutions and journals, such as the University of California system and Antimicrobial Agents and Chemotherapy, have made significant contributions to advancing MRSA bacteremia research. Their abundance of research resources and investment in public health (Fan et al., 2017) have enabled them to maintain a leading position in combating MRSA bacteremia and other antibiotic-resistant BSIs.

From bibliometric analysis, European countries like Germany and the Netherlands stood out in international collaboration, particularly with their high percentage of multi-country publications. The United Kingdom, although producing fewer publications, maintained a close research network with other countries, underscoring its importance in advancing global MRSA bacteremia research. These international collaborations not only help in the dissemination and sharing of knowledge but also strengthen the synergy among countries in effectively addressing the global challenge of MRSA BSIs.

Regarding research institutions, top global institutions have been particularly prominent in both output and influence. For example, the University of California system’s significant contribution to MRSA bacteremia research is reflected in its 116 publications and 3,217 total citations, ranking first in both metrics. The University of Texas system and Harvard University also performed exceptionally in publication volume and citation impact, indicating their pivotal role in advancing research. However, in recent years, as Asian countries have risen in research capabilities, particularly China’s Zhejiang University, which has seen rapid growth in MRSA bacteremia research, these countries have begun to take a more dominant role in the field. Although there is strong cooperation among research institutions within the same country, particularly among U.S. institutions, cross-national collaboration remains relatively limited. This lack of collaboration might hinder the overall progress in MRSA BSI research. Therefore, we strongly recommend strengthening international collaborations between research institutions from different countries to accelerate global research progress on MRSA BSIs.

Compared to other research on antimicrobial infections, studies on MRSA bacteremia are not only concentrated in developed countries but also show an increasing trend of global collaboration. With advancements in technology and resource sharing, more research outputs from low- and middle-income countries are expected to emerge in the future. These countries often face a higher risk of MRSA BSIs (Mohamad Farook et al., 2022), yet research resources are relatively scarce. Strengthening global scientific cooperation and resource sharing can promote MRSA BSI research in these regions and provide a more diverse perspective and data support for the global fight against MRSA bacteremia.

In terms of journals, Antimicrobial Agents and Chemotherapy has published the most studies on MRSA BSIs, far exceeding other academic journals. Additionally, Infection Control and Hospital Epidemiology also leads in both publication and citation counts in this field, demonstrating its significant influence in MRSA BSI research. We also found that most of the top 10 journals are focused on infection and antimicrobial fields, such as the Journal of Antimicrobial Chemotherapy and Infection Control and Hospital Epidemiology. This indicates that current research is largely centered on clinical treatment and infection control (Köck et al., 2010; Kullar et al., 2016), which holds substantial practical significance for improving the diagnosis and treatment of MRSA bacteremia.

From the perspective of authors, Fowler VG from the United States has published the most articles on MRSA BSIs. Rybak MJ, also from the United States, ranks first among co-authors, highlighting his prominent position in this research field. Lodise TP, another American author, has the most citations in this field, exploring various aspects of MRSA’s multidrug resistance and treatment strategies. He and his team have also summarized research related to the epidemiology (Tong et al., 2020; Turner et al., 2019), transmission (David et al., 2014), genetic diversity (Kanafani and Fowler, 2006), as well as monitoring and treatment (Holland et al., 2014) of MRSA BSIs. They have emphasized that although the incidence of MRSA has recently declined in some regions, MRSA still poses a formidable clinical threat (Rasmussen et al., 2011), with morbidity and mortality rates remaining persistently high.

### 4.2 Research hotspots

One of the key roles of bibliometrics is to process and analyze large amounts of data, providing researchers with information about research trends (Xiao et al., 2017). Analyzing frequently occurring keywords can reveal changing trends and prominent themes, which are important for understanding the evolution of this academic field. MRSA BSIs are a major challenge for global health systems. They are typically associated with high mortality, high hospitalization rates, and complex treatment plans (Alhurayri et al., 2021; Datta and Huang, 2008). As a result, current research hotspots focus on several key areas, including antibiotic resistance mechanisms, new diagnostic tools, hospital infection control strategies, and the impact of COVID-19 on MRSA bacteremia. In-depth analysis of these research hotspots helps better understand the progress in MRSA BSI research and predicts future developments.

#### 4.2.1 Current research hotspots

##### 4.2.1.1 Antibiotic resistance and development of treatment methods

Antibiotic resistance is a central challenge in the treatment of MRSA bacteremia. With the increasing resistance of MRSA to methicillin and other commonly used antibiotics (such as vancomycin), treating MRSA BSIs has become increasingly complex (Hassoun et al., 2017). Current research focuses on understanding the resistance mechanisms in MRSA bacteremia and developing new treatment options (Holubar et al., 2020). In recent years, with advances in genomics, researchers have utilized WGS to analyze genetic variations and resistance genes in MRSA strains. These studies provide valuable references for personalized treatment of MRSA bacteremia.

Studies have shown that MRSA strains have developed increasing resistance to vancomycin, prompting researchers to explore alternative therapies and combination therapies for treating MRSA BSIs (Li et al., 2023; Rose et al., 2021). For example, new antibiotics such as linezolid (Dodds and Hawke, 2009) and daptomycin (Adamu et al., 2024) have shown high efficacy in treating MRSA bacteremia. However, as these drugs are increasingly used, resistance is gradually emerging. Future research will likely focus on exploring more effective combinations of antibiotics or other alternative therapies to maximize treatment outcomes and slow the development of resistance.

In addition to hospital-acquired MRSA (HA-MRSA), the rapid spread of community-acquired MRSA (CA-MRSA) strains has exacerbated the public health challenge of MRSA bacteremia (Del Giudice, 2020). These new MRSA strains pose infection risks even to healthy individuals, further complicating the global issue of antibiotic resistance. In response, researchers are actively developing new treatment strategies to address these emerging strains and reduce the overuse of antibiotics.

##### 4.2.1.2 Diagnostic and rapid detection technology development

Early diagnosis of MRSA bacteremia is crucial for effective treatment. Traditional bacterial culture methods are time-consuming and typically take several days, which can lead to treatment delays and increased mortality risk. Therefore, researchers are developing faster and more accurate diagnostic tools that can identify MRSA bacteremia early and guide treatment.

Molecular diagnostic tools based on PCR technology have been widely used in recent years, capable of detecting MRSA strains (Motoshima et al., 2010) within hours. However, advancements in next-generation sequencing (NGS) and genotyping have significantly improved diagnostic efficiency and accuracy (Miao et al., 2017). These new diagnostic tools can quickly identify MRSA BSIs and determine their resistance genotypes, helping physicians formulate more precise treatment plans.

Artificial intelligence is also being introduced into the diagnosis of MRSA BSIs (Gammel et al., 2021). These technologies analyze vast amounts of genomic and clinical data, enabling rapid detection of patterns associated with MRSA bacteremia and improving the speed and accuracy of diagnosis. The application of AI has great potential in enhancing infection control and personalized treatment, and future AI-driven diagnostic technologies are expected to significantly improve the early detection and treatment of MRSA bacteremia.

##### 4.2.1.3 Infection control in healthcare settings

Hospital-acquired MRSA bacteremia remains a significant challenge in global hospital systems, especially in high-risk areas such as ICUs. To reduce the spread of MRSA in hospital environments, researchers have proposed a series of control strategies, including universal screening, patient isolation, hand hygiene management, and environmental disinfection.

Decolonization treatments (e.g., using mupirocin) have been shown to be effective (Poyraz et al., 2022) in reducing the nasal carriage of MRSA strains in high-risk patients. However, long-term use of these drugs may lead to resistance, so future research needs to balance the effectiveness of decolonization treatments with the risk of resistance. Additionally, hospital management policies play a critical role in reducing the transmission of MRSA bacteremia. Studies have shown that implementing strict infection control policies can effectively lower MRSA infection rates (Bertrand et al., 2012) in hospitals.

##### 4.2.1.4 Impact of COVID-19 on MRSA research

The COVID-19 pandemic has exacerbated the risk of bacterial infections in hospital environments, particularly the high incidence of MRSA bacteremia in severe COVID-19 patients. This has drawn researchers’ attention to the management (Tabah and Laupland, 2022) of bacterial and viral co-infections. COVID-19 patients, due to their compromised immune systems (Jeon et al., 2022), are more susceptible to bacterial co-infections, with MRSA bacteremia being a common complication, further complicating treatment.

Research on co-infections of COVID-19 and MRSA has become one of the current hotspots. Studies show that the widespread use of antibiotics during the pandemic has exacerbated the problem of resistance (Jeon et al., 2022), raising the bar for future hospital infection control measures. By deeply studying co-infections of COVID-19 and MRSA, scientists will be better prepared to manage similar co-infection issues in future pandemics and improve current infection control strategies.

#### 4.2.2 Future research hotspots

##### 4.2.2.1 Genomic evolution and personalized medicine

In the future, the application of genomics technology (Dyzenhaus et al., 2023) will continue to drive the progress of MRSA bacteremia research. WGS provides researchers with the opportunity to deeply understand the evolution and resistance development of MRSA strains. By analyzing the genomic data of MRSA strains, researchers can identify key gene mutations (Lindsay, 2013) that lead to resistance and predict the emergence of resistant strains. This information is crucial for developing new treatment strategies, especially for personalized treatment of MRSA bacteremia patients.

In personalized medicine, future research will focus on using genomic data to develop more precise antibiotic treatment plans. By conducting genomic analysis, physicians can select the most appropriate combination of antibiotics based on the specific resistance genes present in the MRSA strain infecting the patient, avoiding the overuse of broad-spectrum antibiotics (Hassoun et al., 2017). Genomics-driven precision treatment not only improves therapeutic efficacy but also helps slow the development of resistance, which is critical for patients with severe infections (Rose et al., 2021).

Moreover, as the cost of genomic sequencing technology (van Belkum and Rochas, 2018) continues to decline, these personalized medical techniques will become increasingly integrated into clinical practice. Physicians will be able to quickly obtain the genomic information of MRSA strains infecting patients and use this data to develop individualized treatment plans. This precision medicine will play a core role in the future management of MRSA bacteremia, helping reduce patient mortality and complications.

##### 4.2.2.2 Integration of artificial intelligence and machine learning

Artificial intelligence and ML (Nigo et al., 2024) will play an increasingly important role in the research and clinical management of MRSA bacteremia. These technologies process vast amounts of genomic and clinical information (Lu et al., 2021), accelerating the early diagnosis of MRSA infections and the prediction of antibiotic resistance. For example, AI algorithms can analyze a patient’s genomic information and medical history to predict their risk of developing MRSA bacteremia and provide personalized treatment suggestions based on the predictions.

In diagnostics, AI can automate the processing of large amounts of laboratory data, quickly identifying MRSA bacteremia patients and providing accurate diagnostic results. AI can also analyze historical case data to predict the evolutionary trends of MRSA strains and the possible future emergence of resistant strains. This predictive ability can help healthcare institutions better manage the use of antibiotics and prevent the spread of resistant strains (Lin et al., 2023; Lu et al., 2021).

Artificial intelligence will also play a role in drug development. By analyzing mutation patterns in MRSA strains, AI can help researchers identify potential new drug targets and accelerate the development of novel antibiotics. This data-driven research approach will significantly improve the efficiency of diagnosing and treating MRSA bacteremia, especially as antibiotic effectiveness declines. The integration of AI will provide strong support for combating resistance (Bueschbell et al., 2022; Liu et al., 2024).

##### 4.2.2.3 Phage therapy

Phage therapy (Strathdee et al., 2023) is an emerging research direction for combating MRSA bacteremia. Bacteriophages are viruses that specifically infect and kill bacteria. They have a high specificity for MRSA strains, making them a promising alternative to antibiotics, particularly in the context of increasing antibiotic resistance. Phage therapy has shown great potential in the treatment of MRSA bacteremia (Plumet et al., 2022), as it can selectively kill MRSA strains without disrupting the body’s normal microbiota.

Although phage therapy has demonstrated good results in laboratory studies, it still faces some challenges in clinical applications. Future research will focus on optimizing the safety and efficacy of phage therapy and exploring its potential combination with traditional antibiotics. For example, by combining phages with existing antibiotics, researchers hope to enhance the treatment effectiveness for MRSA bacteremia while reducing antibiotic use, thus delaying the development of resistance.

Additionally, phage therapy has potential in preventing MRSA infections. Future research may develop phage sprays or coatings to reduce the risk of MRSA transmission in hospital environments. By combining phage therapy with existing infection control measures, future strategies for the prevention and treatment of MRSA bacteremia will become more diverse and effective.

##### 4.2.2.4 Development of MRSA vaccines

The development of MRSA vaccines (Chand et al., 2023) is a key area in the future fight against MRSA bacteremia. Although there is currently no widely available MRSA vaccine, the successful application of mRNA vaccine technology has made the development prospects for MRSA vaccines more optimistic. Researchers are working to develop vaccines that can elicit long-lasting immune responses to prevent the occurrence of MRSA bacteremia, particularly in immunocompromised patients.

A successful MRSA vaccine would help reduce the incidence of MRSA BSIs and lower the reliance on antibiotics, thereby curbing the spread of antibiotic resistance globally (Lindsay, 2007). The use of MRSA vaccines would be especially beneficial for long-term hospitalized patients and high-risk populations, significantly reducing their risk of developing MRSA bacteremia. Future research will focus on optimizing vaccine efficacy and ensuring that it can combat the diversity and rapid evolution of MRSA strains.

With continuous advancements in vaccine technology, MRSA vaccines are expected to enter clinical trials in the coming years and eventually gain approval (Miller et al., 2020). This would be a significant breakthrough in the global fight against MRSA bacteremia, not only significantly reducing infection rates but also effectively controlling antibiotic misuse and delaying the development of resistance.

### 4.3 Limitations

While this study provides valuable insights into the research trends and future directions of MRSA BSIs, it has several limitations. First, the data used in this bibliometric analysis were sourced solely from the WoSCC, potentially omitting relevant publications indexed in other databases such as Scopus or PubMed. This may lead to an incomplete representation of the global research landscape on MRSA BSIs.

## 5 Conclusion

Methicillin-resistant *Staphylococcus aureus* BSIs remain a significant global public health challenge, particularly in the context of increasing antibiotic resistance. Despite notable advances in antimicrobial therapy, rapid diagnostic techniques, and emerging therapies such as phage therapy and vaccine development, further clinical validation and optimization are still needed. Future research directions will increasingly rely on the integration of genomics, AI, and ML to enhance the precision and effectiveness of personalized treatments.

Global collaboration will be a key driver of MRSA BSI research, especially in low- and middle-income countries, where the risk of infection is higher but research resources are relatively limited. By strengthening global cooperation and promoting the sharing of scientific resources, there is hope for effectively curbing the spread of MRSA BSIs worldwide and improving the ability to tackle antibiotic resistance.

## Data Availability

The original contributions presented in this study are included in this article/Supplementary material, further inquiries can be directed to the corresponding author.
